# Supplementary material for: Diagnostic performance of chest computed tomography during the epidemic wave of COVID-19 varied as a function of time since the beginning of the confinement in France
Source: PLoS One. 2020 Nov 23;15(11):e0242840. doi: 10.1371/journal.pone.0242840 (PMC7682866; doi:10.1371/journal.pone.0242840)
Supplement: S1 File — (DOCX) [file pone.0242840.s001.docx]

S1 File. CT-scanning

All scans were performed on a scanner dedicated to COVID-19 patient management within the ER environment (Siemens Somaton Edge) either with or without iodine contrast injection according to the pretest risk of pulmonary embolism. Suspicion of pulmonary embolism was defined either by oxygen dependence requiring nasal O2 at a rate >3l/min, or by the use of a combination of the revised Geneva score and D-dimer above the age-adjusted threshold, leading to a risk considered high. Patients were in supine position and acquisition was performed at full inspiration. The scanning parameters were: slice thickness 80 x 0.625 mm, rotation time 0.28 s, pitch 0.992, field of view 350 mm x 350 mm, matrix 512 x 512, tube voltage = 120 kV average tube current 300 mA (automatic modulation). All images were reconstructed with a slice thickness of 0.625 –1.250 with the same increment.
