# Supplementary material for: Diagnostic performance of chest computed tomography during the epidemic wave of COVID-19 varied as a function of time since the beginning of the confinement in France
Source: PLoS One. 2020 Nov 23;15(11):e0242840. doi: 10.1371/journal.pone.0242840 (PMC7682866; doi:10.1371/journal.pone.0242840)
Supplement: S2 File — (DOCX) [file pone.0242840.s002.docx]

S2 File. RT-PCR

Nasopharyngeal swab samples were performed to allow detection of SARS-CoV-2 RNA by one of the two following real time RT-PCR assays : the RealStar SARS-CoV-2 RT-PCR kit 1.0 (Altona Diagnostics, Germany) on LightCycler 480 instrument (Roche Diagnostics, France) after viral nucleic acid extraction with NucliSens easyMAG system (BioMérieux, France) or using the fully-automated cobas SARS-CoV-2 test on the cobas 6800 system (Roche Diagnostics, France) according to the manufacturer’s instructions.
