# Supplementary material for: Diagnostic performance of chest computed tomography during the epidemic wave of COVID-19 varied as a function of time since the beginning of the confinement in France
Source: PLoS One. 2020 Nov 23;15(11):e0242840. doi: 10.1371/journal.pone.0242840 (PMC7682866; doi:10.1371/journal.pone.0242840)
Supplement: S3 File — (DOCX) [file pone.0242840.s003.docx]

S3 File. Statistical analysis

The statistical analysis was performed using SPSS version 21.0 (SPSS Inc. Chicago, IL). Continuous variables were displayed as mean ± standard deviation and categorical variables were reported as counts and percentages. In order to calculate CT accuracy for the diagnosis of COVID-19, both CT report statements “suggestive of COVID-19” and “suggestive of a combination of COVID-19 with another lung disease” were considered positive for COVID-19. The statements “suggestive of another lung disease” and “normal” were considered negative for COVID-19.
